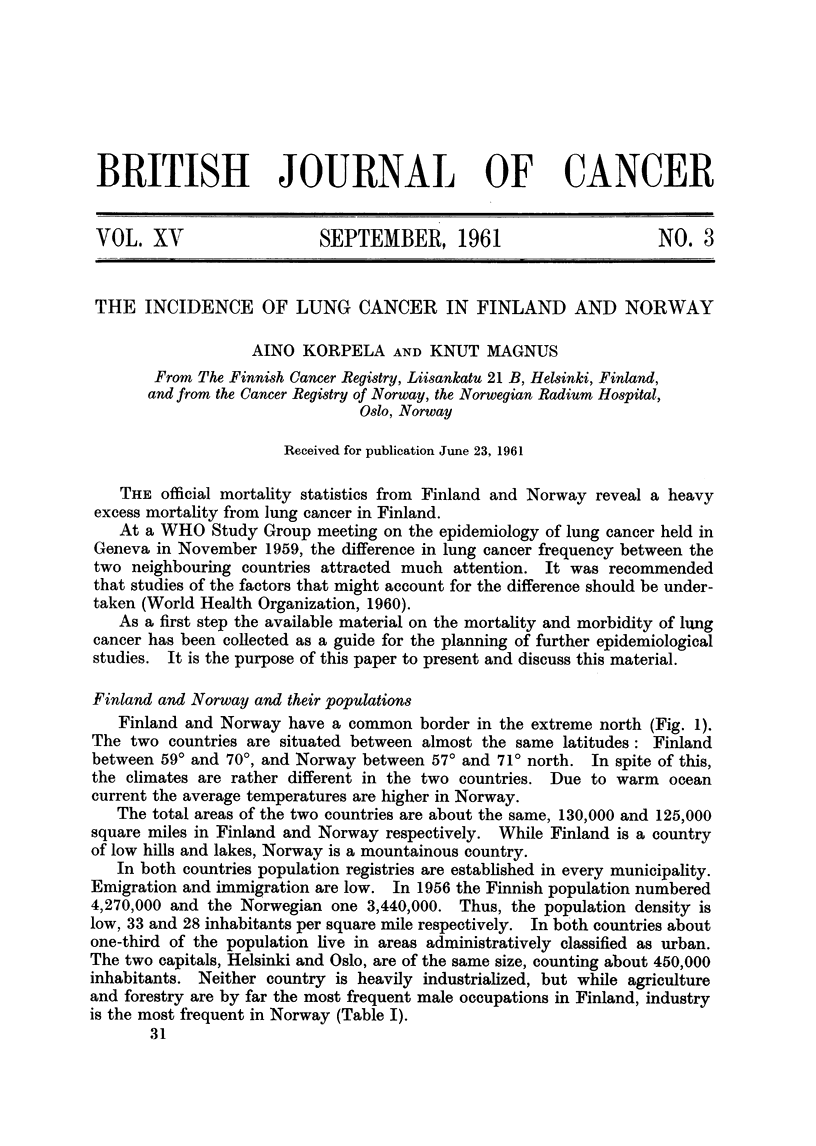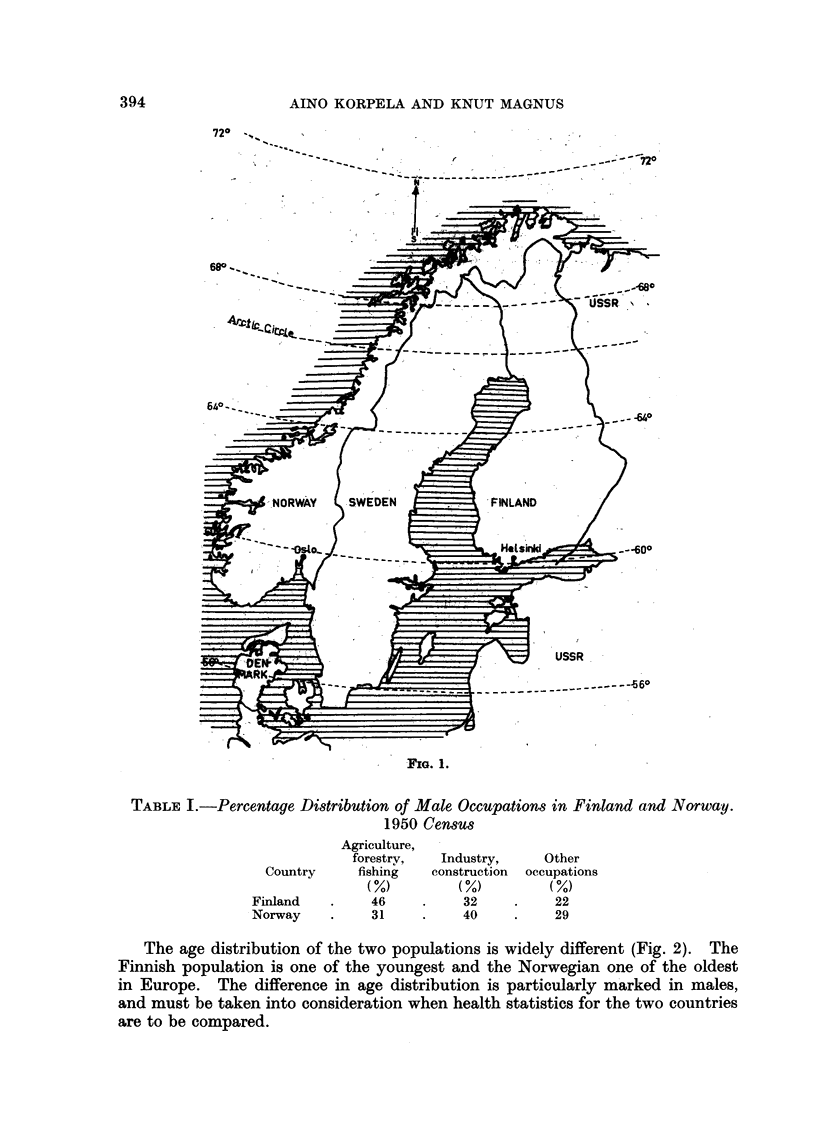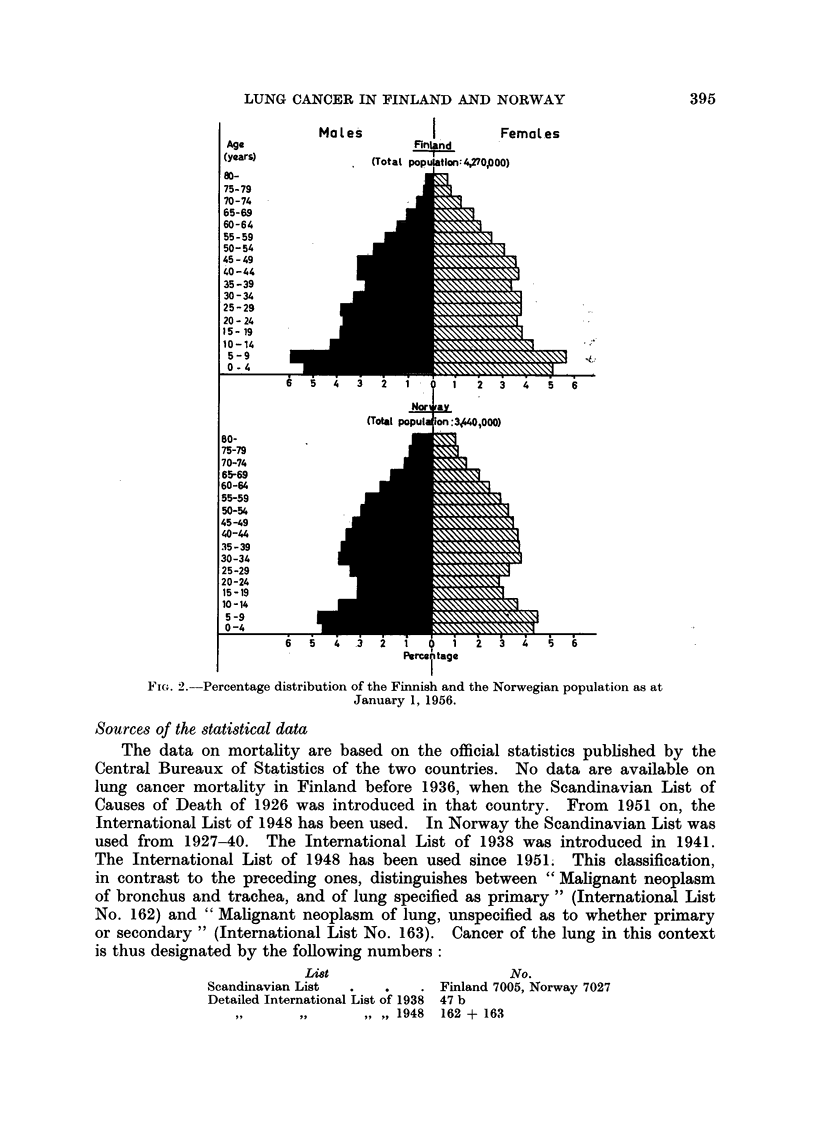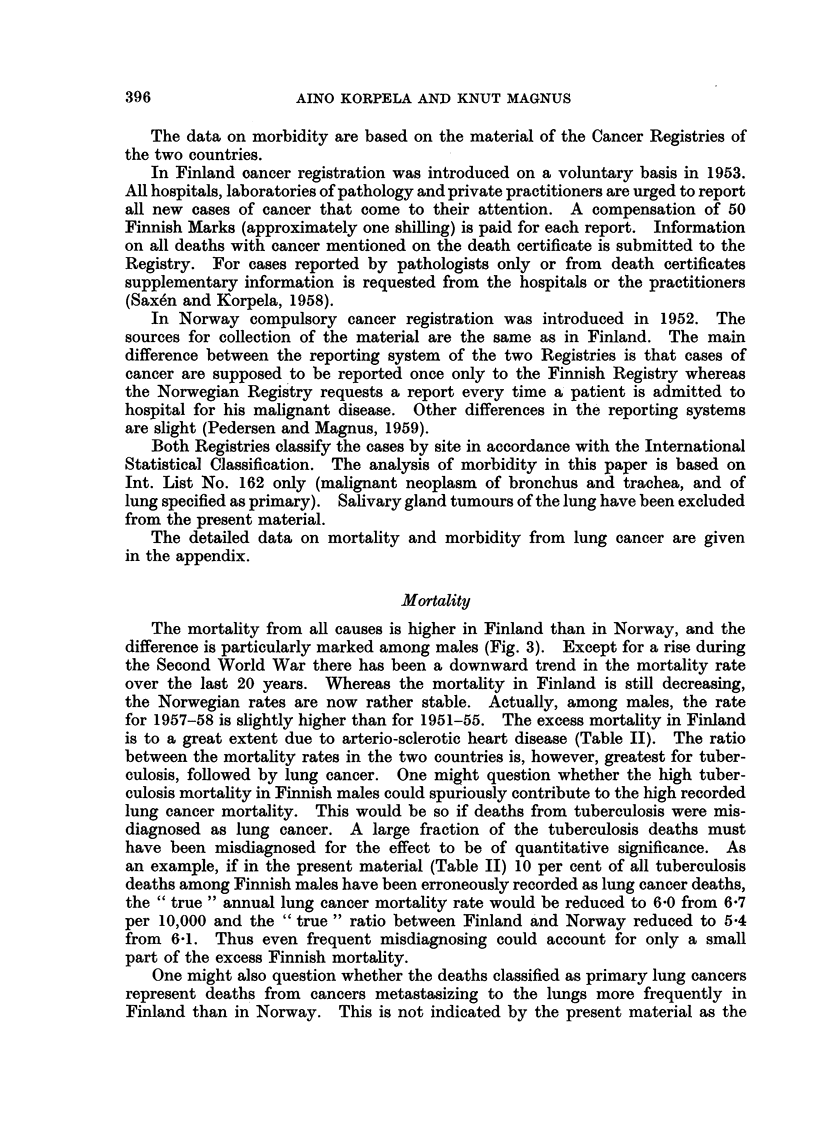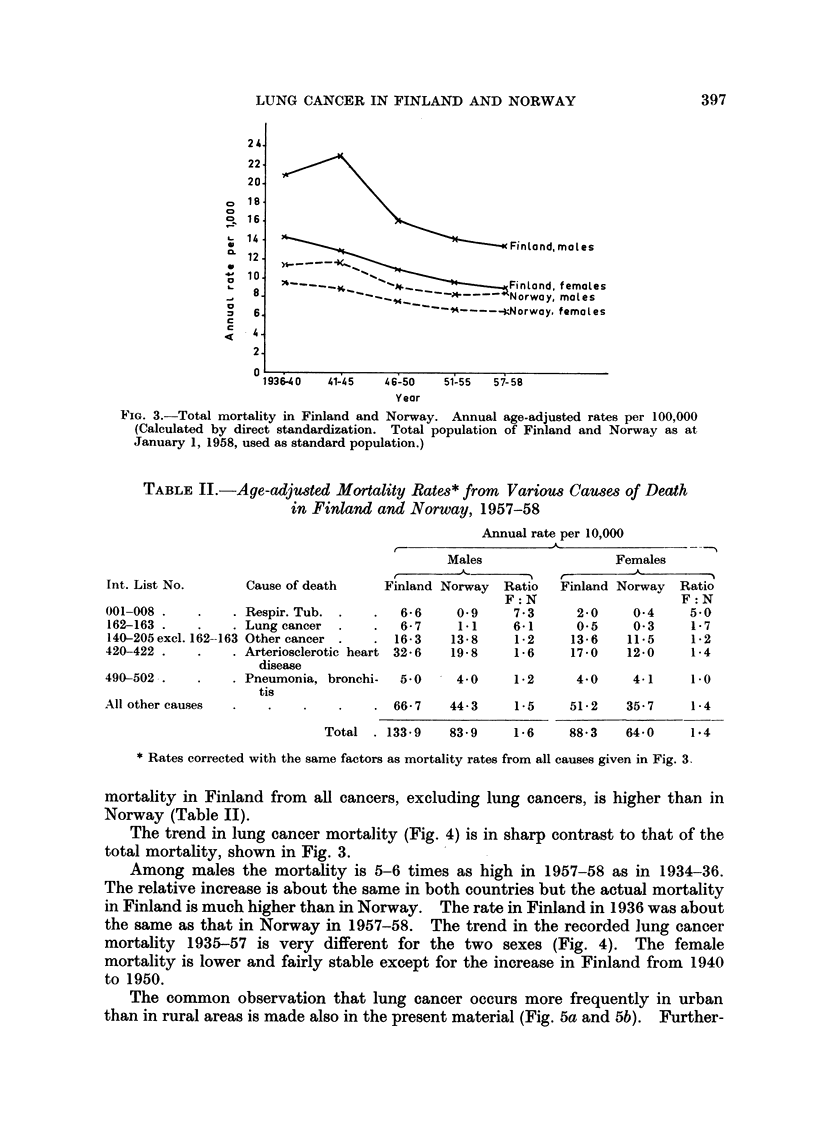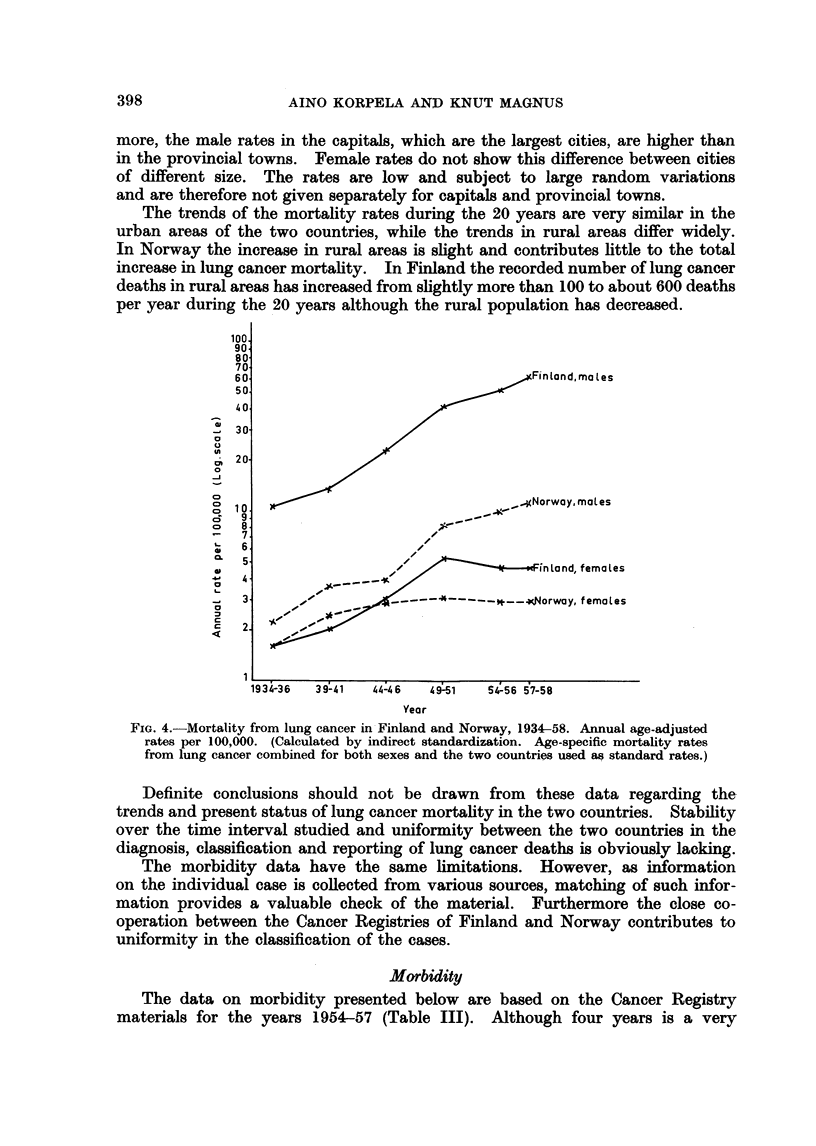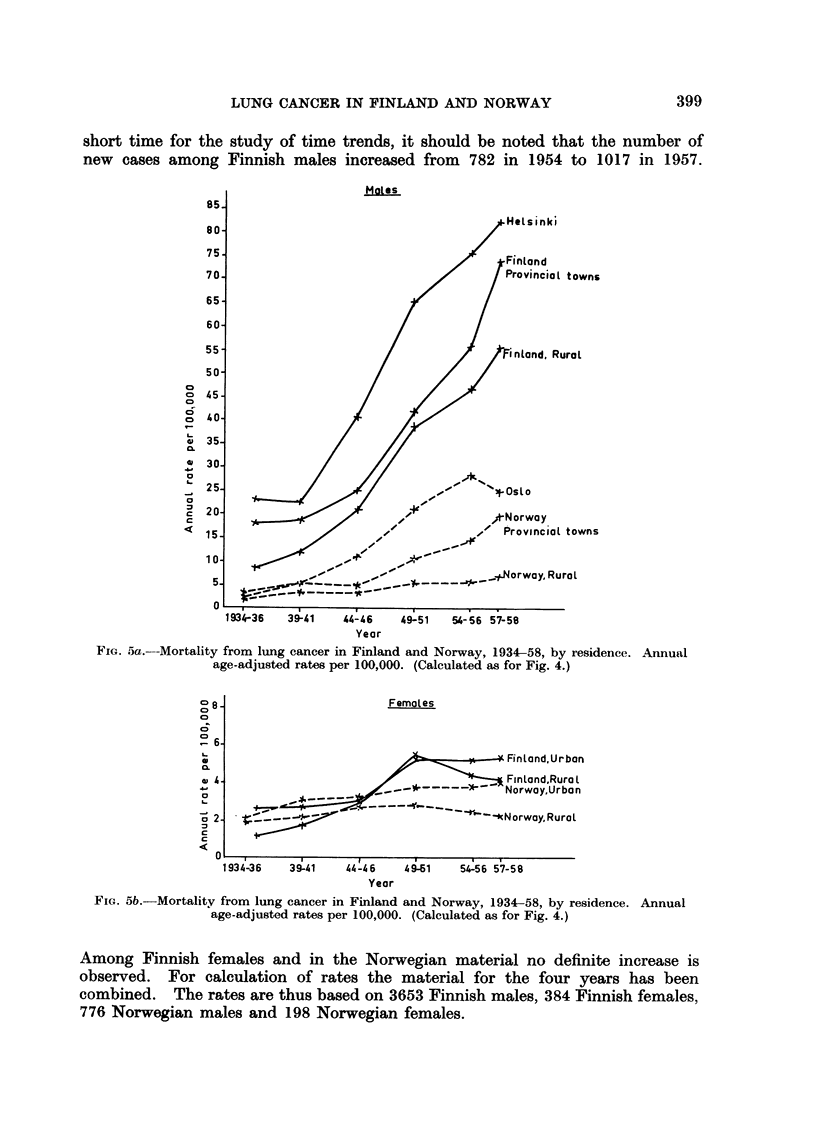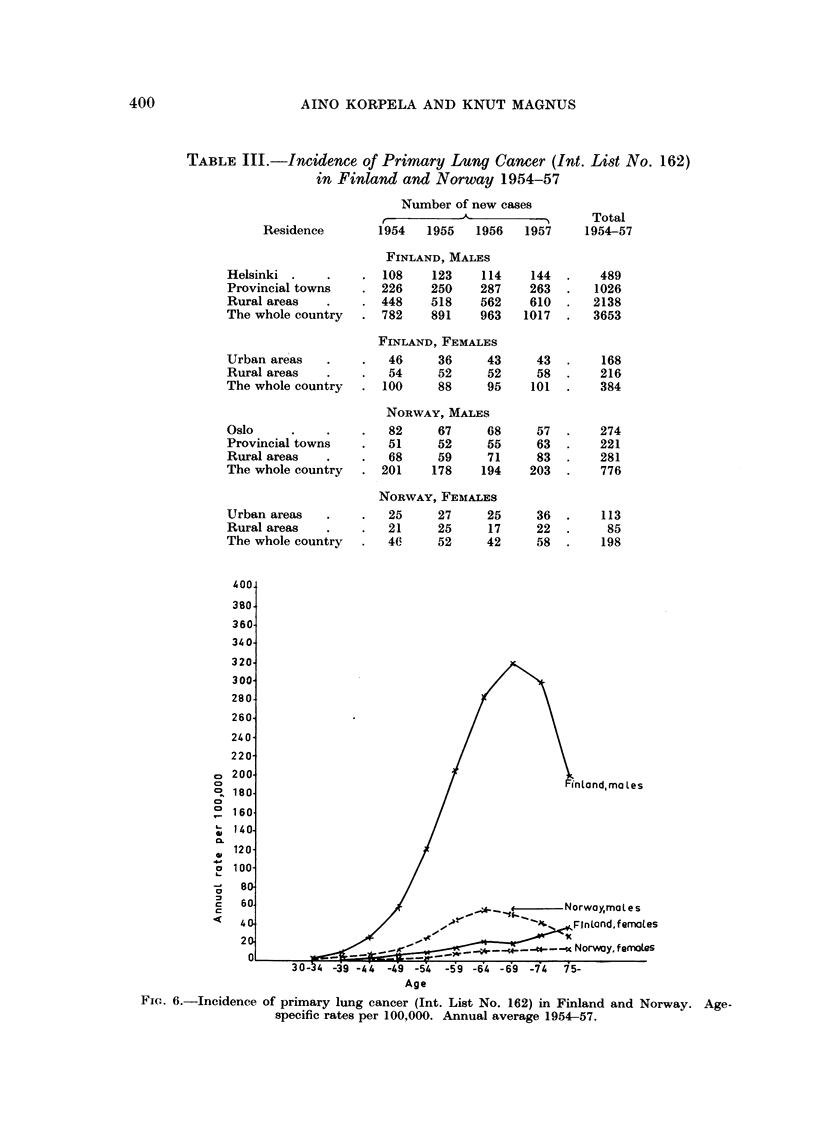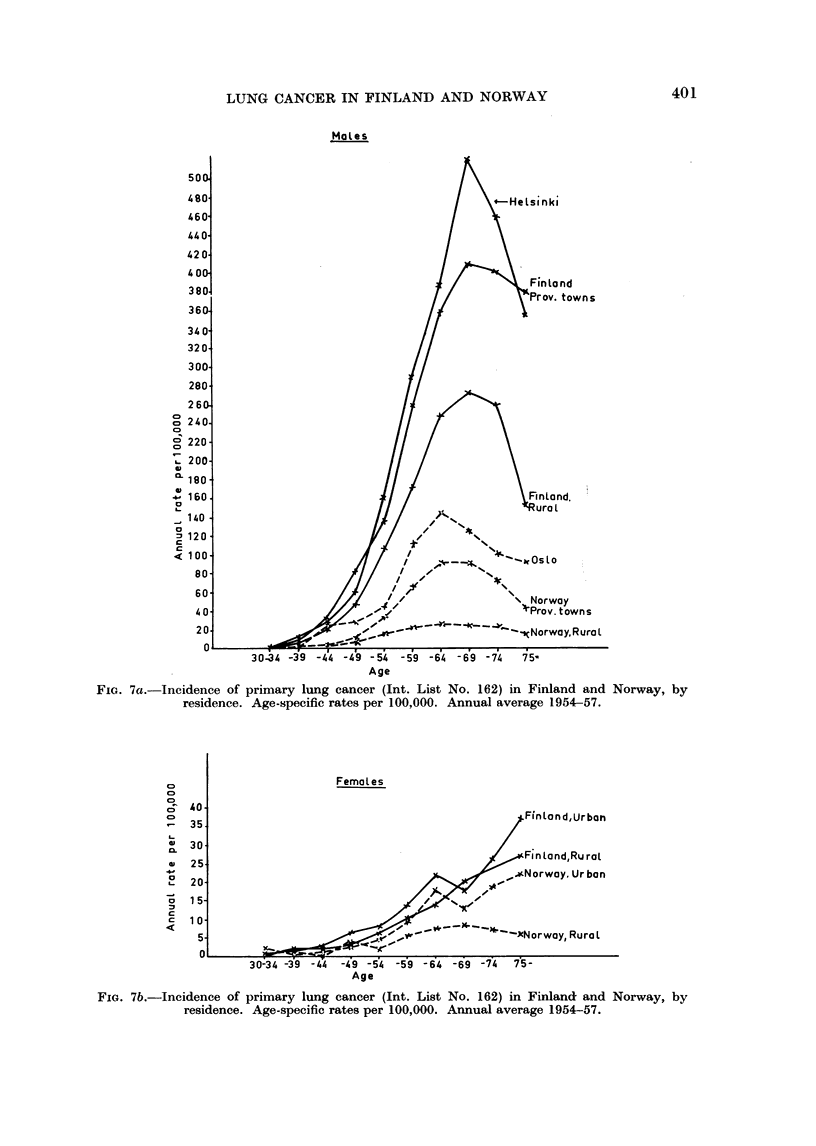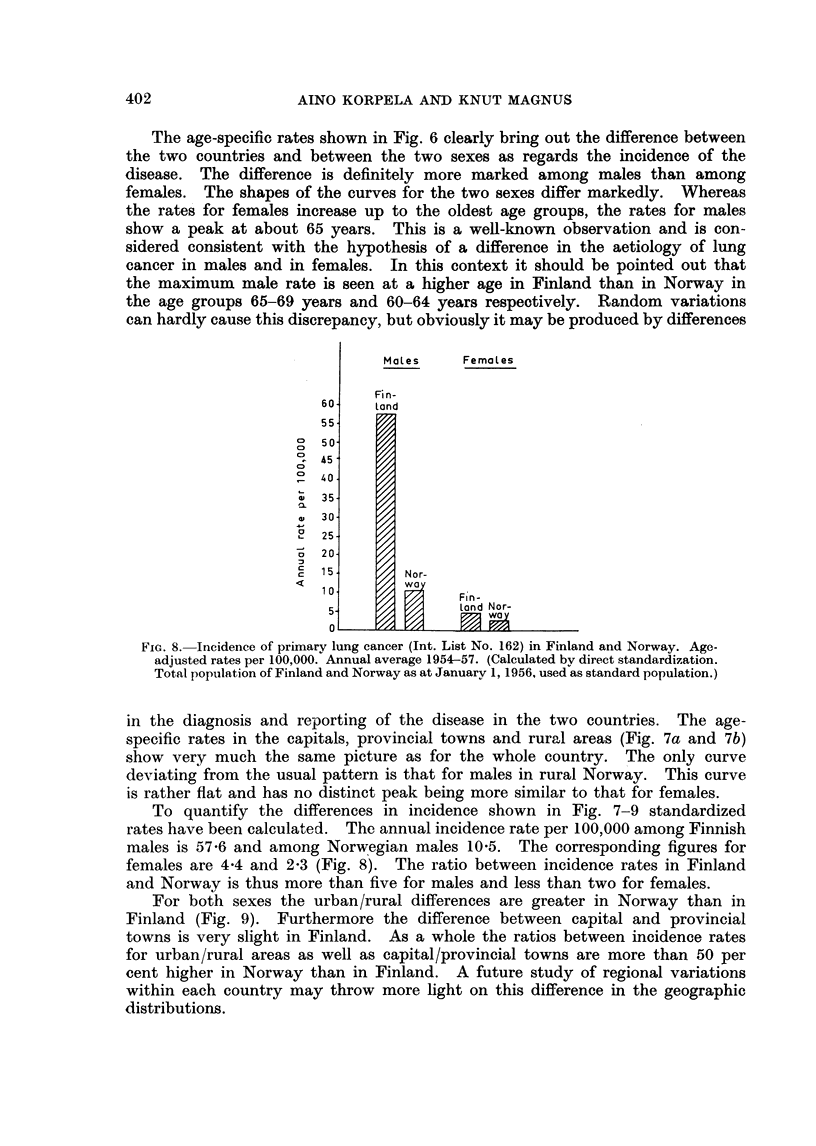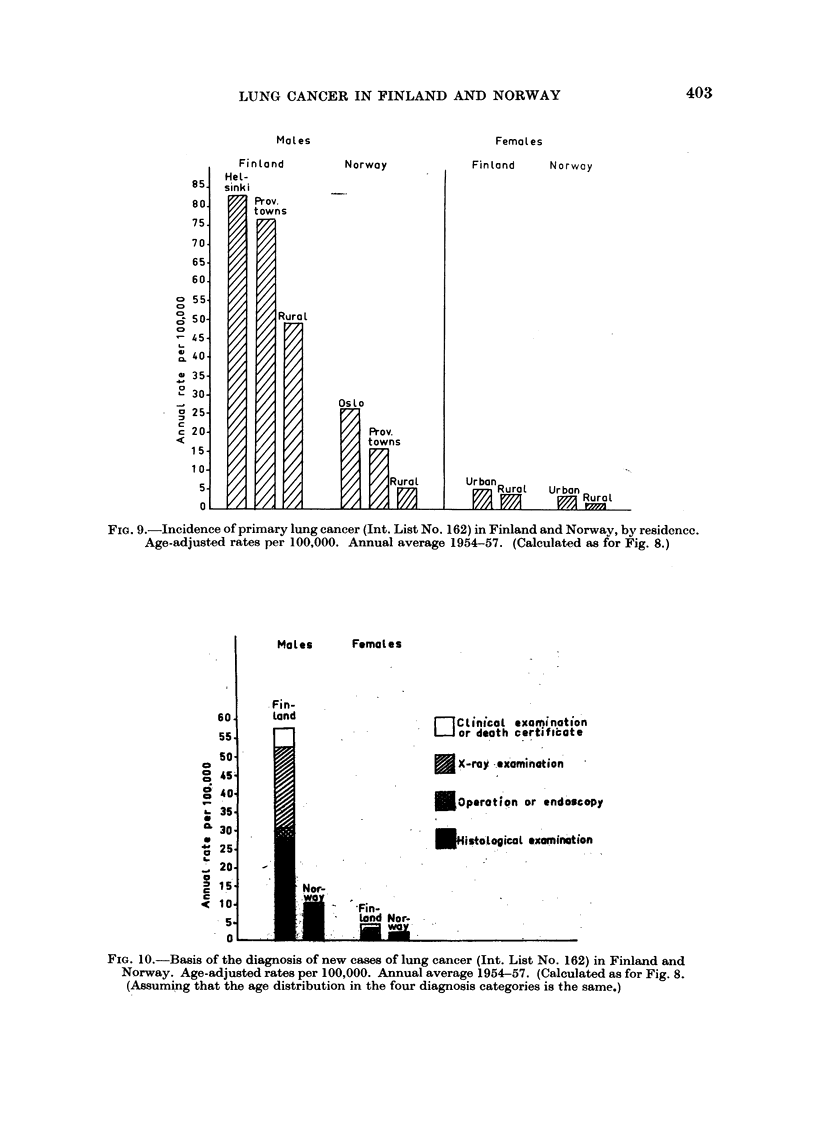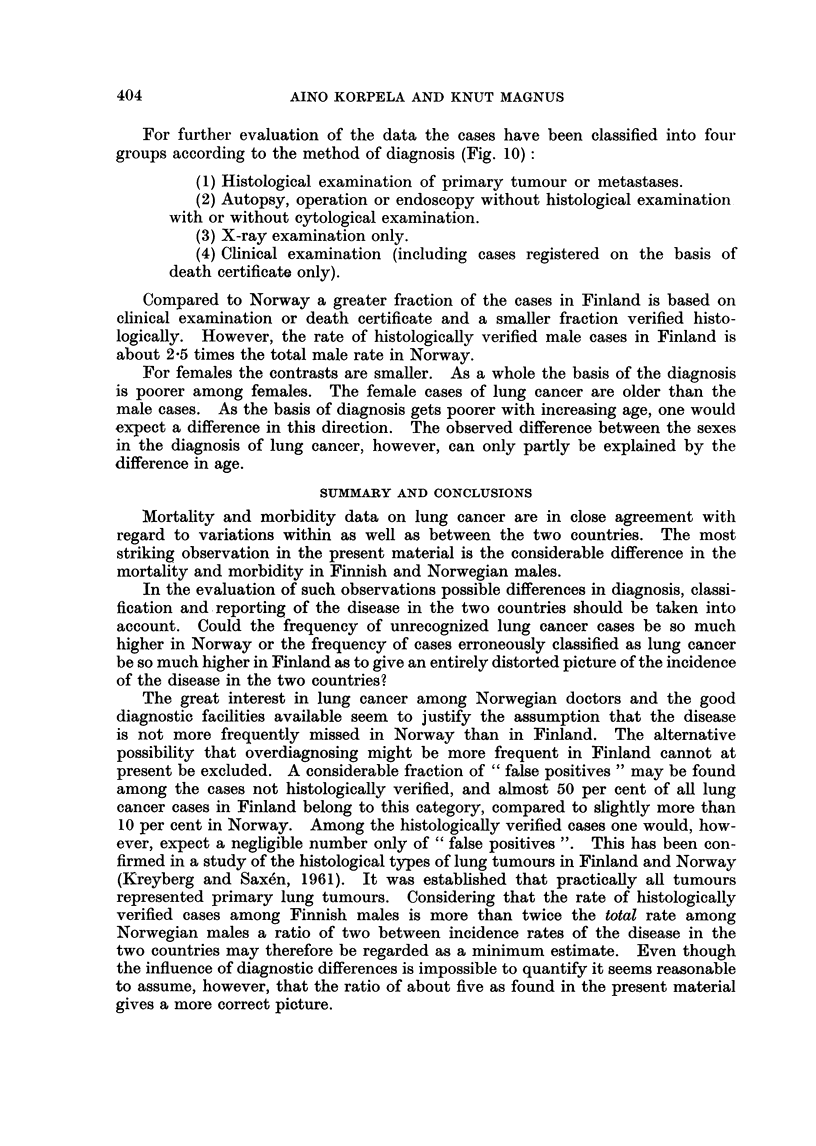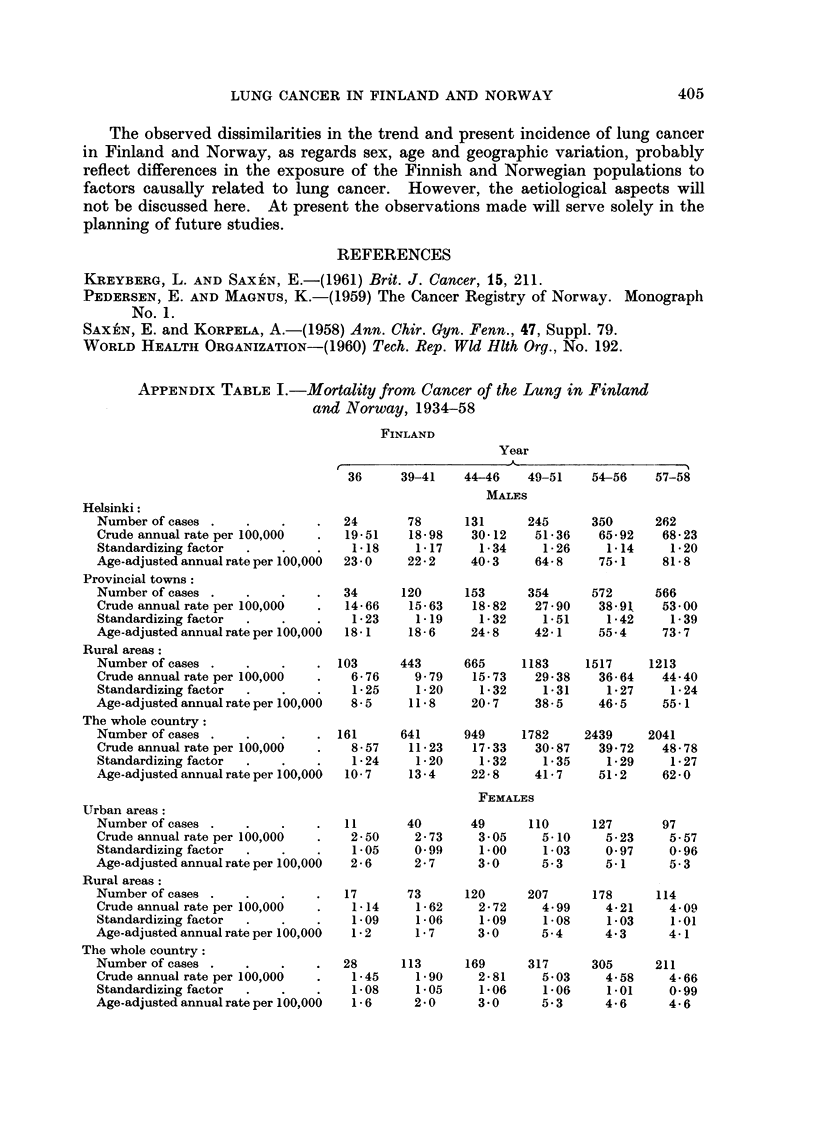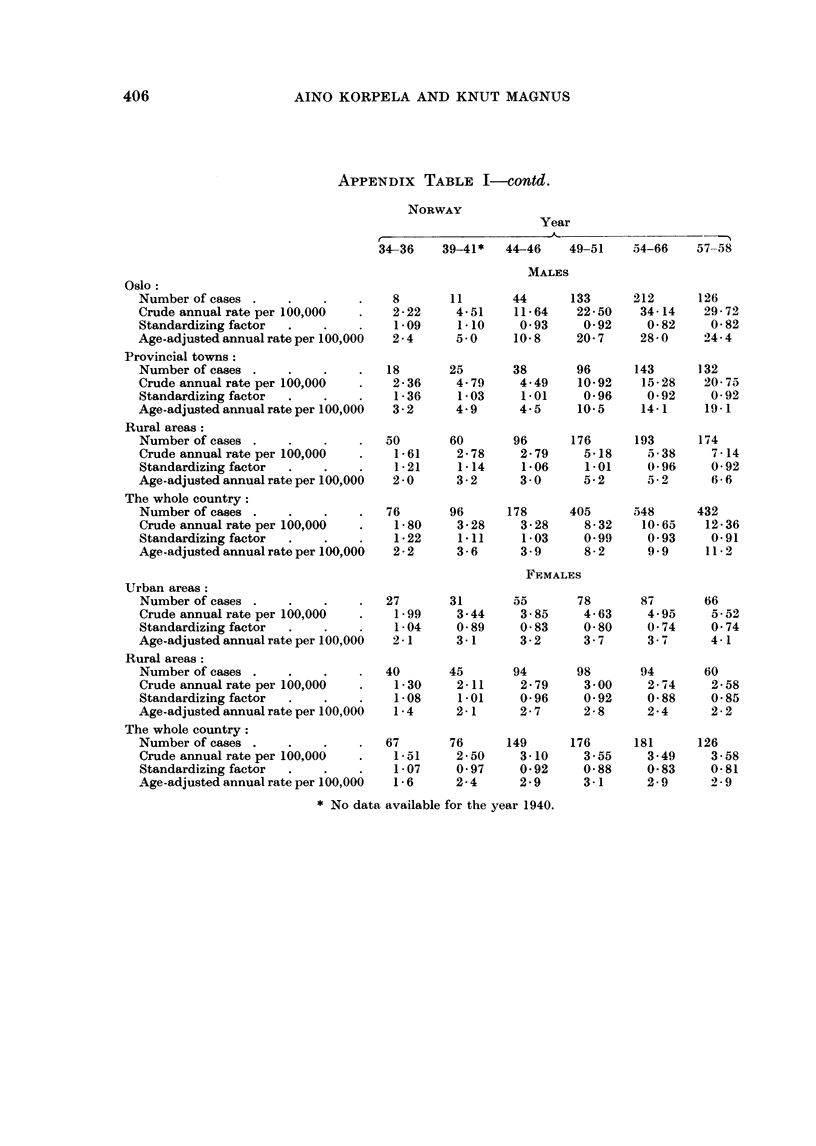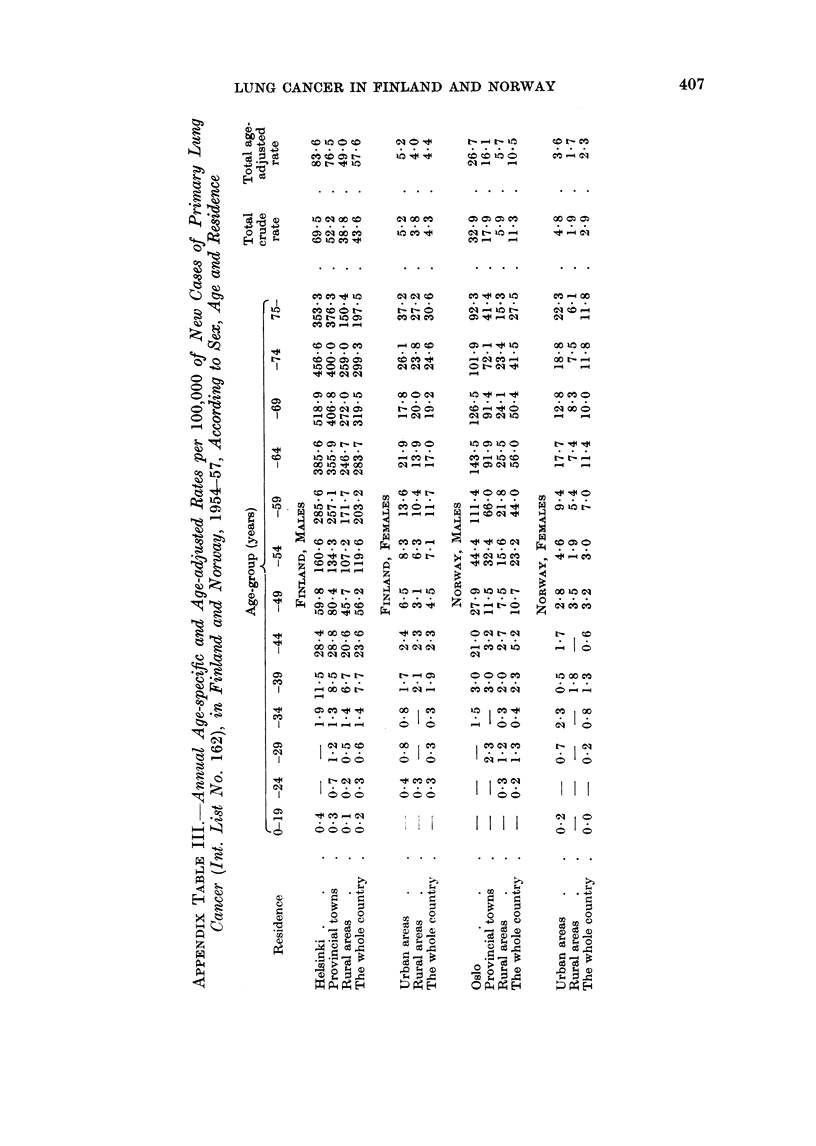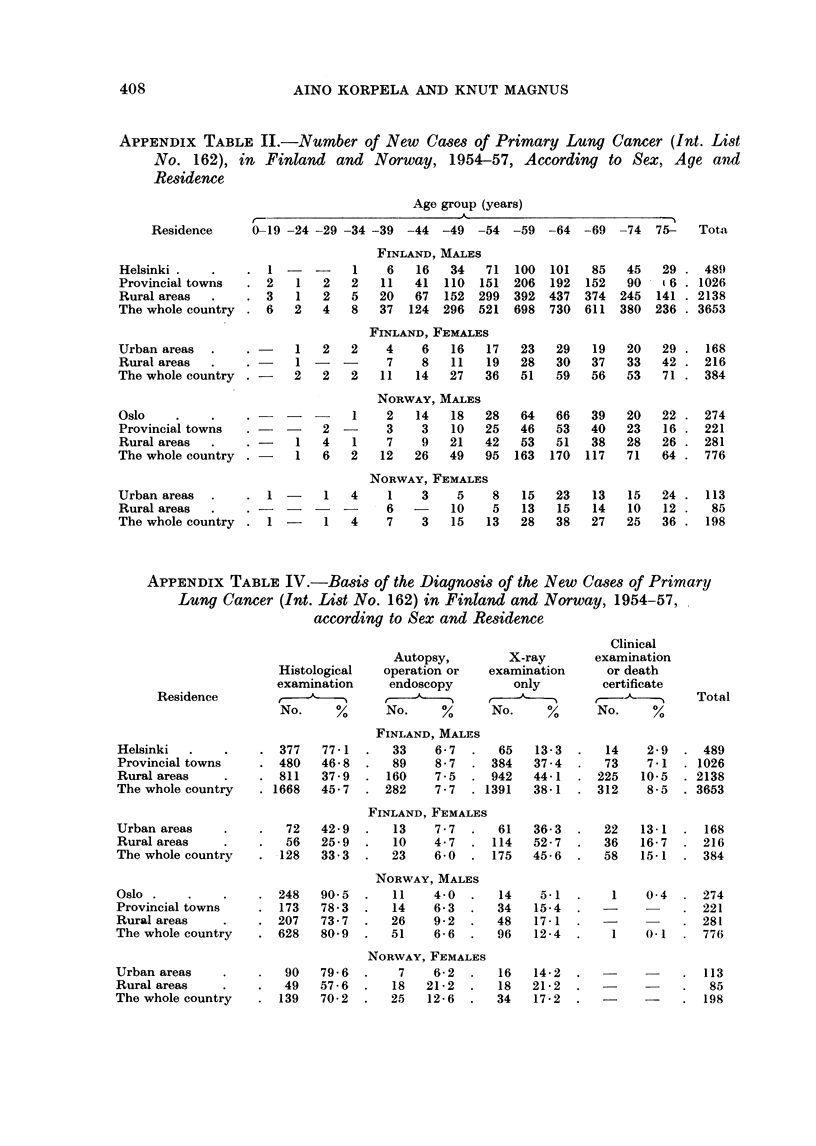# The Incidence of Lung Cancer in Finland and Norway

**DOI:** 10.1038/bjc.1961.50

**Published:** 1961-09

**Authors:** Aino Korpela, Knut Magnus


### BRITISH JOURNAL OF CANCER

VOL. XV SEPTEMBER, 1961 NO. 3

### THE INCIDENCE OF LUNG CANCER IN FINLAND AND NORWAY

AINO KORPELA AND KNUT MAGNUS

*From The Finnish Cancer Registry, Liisankatu 21 B, Helsinki, Finland, and from the Cancer Registry of Norway, the Norwegian Radium Hospital, Oslo, Norway*

Received for publication June 23, 1961

THE official mortality statistics from Finland and Norway reveal a heavy excess mortality from lung cancer in Finland.

At a WHO Study Group meeting on the epidemiology of lung cancer held in Geneva in November 1959, the difference in lung cancer frequency between the two neighbouring countries attracted much attention. It was recommended that studies of the factors that might account for the difference should be undertaken (World Health Organization, 1960).

As a first step the available material on the mortality and morbidity of lung cancer has been collected as a guide for the planning of further epidemiological studies. It is the purpose of this paper to present and discuss this material.

*Finland and Norway and their populations*

Finland and Norway have a common border in the extreme north (Fig. 1). The two countries are situated between almost the same latitudes: Finland between 59° and 70°, and Norway between 57° and 71° north. In spite of this, the climates are rather different in the two countries. Due to warm ocean current the average temperatures are higher in Norway.

The total areas of the two countries are about the same, 130,000 and 125,000 square miles in Finland and Norway respectively. While Finland is a country of low hills and lakes, Norway is a mountainous country.

In both countries population registries are established in every municipality. Emigration and immigration are low. In 1956 the Finnish population numbered 4,270,000 and the Norwegian one 3,440,000. Thus, the population density is low, 33 and 28 inhabitants per square mile respectively. In both countries about one-third of the population live in areas administratively classified as urban. The two capitals, Helsinki and Oslo, are of the same size, counting about 450,000 inhabitants. Neither country is heavily industrialized, but while agriculture and forestry are by far the most frequent male occupations in Finland, industry is the most frequent in Norway (Table I).

FIG. 1.

TABLE I.—*Percentage Distribution of Male Occupations in Finland and Norway. 1950 Census*

| Country | Agriculture, forestry, fishing (%) | Industry, construction (%) | Other occupations (%) |
|---|---|---|---|
| Finland | 46 | 32 | 22 |
| Norway | 31 | 40 | 29 |

The age distribution of the two populations is widely different (Fig. 2). The Finnish population is one of the youngest and the Norwegian one of the oldest in Europe. The difference in age distribution is particularly marked in males, and must be taken into consideration when health statistics for the two countries are to be compared.

FIG. 2.—Percentage distribution of the Finnish and the Norwegian population as at January 1, 1956.

*Sources of the statistical data*

The data on mortality are based on the official statistics published by the Central Bureaux of Statistics of the two countries. No data are available on lung cancer mortality in Finland before 1936, when the Scandinavian List of Causes of Death of 1926 was introduced in that country. From 1951 on, the International List of 1948 has been used. In Norway the Scandinavian List was used from 1927–40. The International List of 1938 was introduced in 1941. The International List of 1948 has been used since 1951. This classification, in contrast to the preceding ones, distinguishes between " Malignant neoplasm of bronchus and trachea, and of lung specified as primary " (International List No. 162) and " Malignant neoplasm of lung, unspecified as to whether primary or secondary " (International List No. 163). Cancer of the lung in this context is thus designated by the following numbers :

| *List* | *No.* |
|---|---|
| Scandinavian List . . . | Finland 7005, Norway 7027 |
| Detailed International List of 1938 | 47 b |
| ,, ,, ,, ,, 1948 | 162 + 163 |

The data on morbidity are based on the material of the Cancer Registries of the two countries.

In Finland cancer registration was introduced on a voluntary basis in 1953. All hospitals, laboratories of pathology and private practitioners are urged to report all new cases of cancer that come to their attention. A compensation of 50 Finnish Marks (approximately one shilling) is paid for each report. Information on all deaths with cancer mentioned on the death certificate is submitted to the Registry. For cases reported by pathologists only or from death certificates supplementary information is requested from the hospitals or the practitioners (Saxén and Korpela, 1958).

In Norway compulsory cancer registration was introduced in 1952. The sources for collection of the material are the same as in Finland. The main difference between the reporting system of the two Registries is that cases of cancer are supposed to be reported once only to the Finnish Registry whereas the Norwegian Registry requests a report every time a patient is admitted to hospital for his malignant disease. Other differences in the reporting systems are slight (Pedersen and Magnus, 1959).

Both Registries classify the cases by site in accordance with the International Statistical Classification. The analysis of morbidity in this paper is based on Int. List No. 162 only (malignant neoplasm of bronchus and trachea, and of lung specified as primary). Salivary gland tumours of the lung have been excluded from the present material.

The detailed data on mortality and morbidity from lung cancer are given in the appendix.

### *Mortality*

The mortality from all causes is higher in Finland than in Norway, and the difference is particularly marked among males (Fig. 3). Except for a rise during the Second World War there has been a downward trend in the mortality rate over the last 20 years. Whereas the mortality in Finland is still decreasing, the Norwegian rates are now rather stable. Actually, among males, the rate for 1957–58 is slightly higher than for 1951–55. The excess mortality in Finland is to a great extent due to arterio-sclerotic heart disease (Table II). The ratio between the mortality rates in the two countries is, however, greatest for tuberculosis, followed by lung cancer. One might question whether the high tuberculosis mortality in Finnish males could spuriously contribute to the high recorded lung cancer mortality. This would be so if deaths from tuberculosis were misdiagnosed as lung cancer. A large fraction of the tuberculosis deaths must have been misdiagnosed for the effect to be of quantitative significance. As an example, if in the present material (Table II) 10 per cent of all tuberculosis deaths among Finnish males have been erroneously recorded as lung cancer deaths, the " true " annual lung cancer mortality rate would be reduced to 6·0 from 6·7 per 10,000 and the " true " ratio between Finland and Norway reduced to 5·4 from 6·1. Thus even frequent misdiagnosing could account for only a small part of the excess Finnish mortality.

One might also question whether the deaths classified as primary lung cancers represent deaths from cancers metastasizing to the lungs more frequently in Finland than in Norway. This is not indicated by the present material as the

FIG. 3.—Total mortality in Finland and Norway. Annual age-adjusted rates per 100,000 (Calculated by direct standardization. Total population of Finland and Norway as at January 1, 1958, used as standard population.)

TABLE II.—*Age-adjusted Mortality Rates* from Various Causes of Death in Finland and Norway*, 1957–58

| Int. List No. | Cause of death | Males: Finland | Males: Norway | Males: Ratio F : N | Females: Finland | Females: Norway | Females: Ratio F : N |
|---|---|---|---|---|---|---|---|
| | | Annual rate per 10,000 | | | | | |
| 001–008 . . . | Respir. Tub. . . | 6·6 | 0·9 | 7·3 | 2·0 | 0·4 | 5·0 |
| 162–163 . . . | Lung cancer . . | 6·7 | 1·1 | 6·1 | 0·5 | 0·3 | 1·7 |
| 140–205 excl. 162–163 | Other cancer . . | 16·3 | 13·8 | 1·2 | 13·6 | 11·5 | 1·2 |
| 420–422 . . . | Arteriosclerotic heart disease | 32·6 | 19·8 | 1·6 | 17·0 | 12·0 | 1·4 |
| 490–502 . . . | Pneumonia, bronchitis | 5·0 | 4·0 | 1·2 | 4·0 | 4·1 | 1·0 |
| All other causes | . . . . . | 66·7 | 44·3 | 1·5 | 51·2 | 35·7 | 1·4 |
| | Total . | 133·9 | 83·9 | 1·6 | 88·3 | 64·0 | 1·4 |

* Rates corrected with the same factors as mortality rates from all causes given in Fig. 3.

mortality in Finland from all cancers, excluding lung cancers, is higher than in Norway (Table II).

The trend in lung cancer mortality (Fig. 4) is in sharp contrast to that of the total mortality, shown in Fig. 3.

Among males the mortality is 5–6 times as high in 1957–58 as in 1934–36. The relative increase is about the same in both countries but the actual mortality in Finland is much higher than in Norway. The rate in Finland in 1936 was about the same as that in Norway in 1957–58. The trend in the recorded lung cancer mortality 1935–57 is very different for the two sexes (Fig. 4). The female mortality is lower and fairly stable except for the increase in Finland from 1940 to 1950.

The common observation that lung cancer occurs more frequently in urban than in rural areas is made also in the present material (Fig. 5*a* and 5*b*). Furthermore, the male rates in the capitals, which are the largest cities, are higher than in the provincial towns. Female rates do not show this difference between cities of different size. The rates are low and subject to large random variations and are therefore not given separately for capitals and provincial towns.

The trends of the mortality rates during the 20 years are very similar in the urban areas of the two countries, while the trends in rural areas differ widely. In Norway the increase in rural areas is slight and contributes little to the total increase in lung cancer mortality. In Finland the recorded number of lung cancer deaths in rural areas has increased from slightly more than 100 to about 600 deaths per year during the 20 years although the rural population has decreased.

FIG. 4.—Mortality from lung cancer in Finland and Norway, 1934–58. Annual age-adjusted rates per 100,000. (Calculated by indirect standardization. Age-specific mortality rates from lung cancer combined for both sexes and the two countries used as standard rates.)

Definite conclusions should not be drawn from these data regarding the trends and present status of lung cancer mortality in the two countries. Stability over the time interval studied and uniformity between the two countries in the diagnosis, classification and reporting of lung cancer deaths is obviously lacking.

The morbidity data have the same limitations. However, as information on the individual case is collected from various sources, matching of such information provides a valuable check of the material. Furthermore the close cooperation between the Cancer Registries of Finland and Norway contributes to uniformity in the classification of the cases.

### *Morbidity*

The data on morbidity presented below are based on the Cancer Registry materials for the years 1954–57 (Table III). Although four years is a very short time for the study of time trends, it should be noted that the number of new cases among Finnish males increased from 782 in 1954 to 1017 in 1957.

FIG. 5a.—Mortality from lung cancer in Finland and Norway, 1934–58, by residence. Annual age-adjusted rates per 100,000. (Calculated as for Fig. 4.)

FIG. 5b.—Mortality from lung cancer in Finland and Norway, 1934–58, by residence. Annual age-adjusted rates per 100,000. (Calculated as for Fig. 4.)

Among Finnish females and in the Norwegian material no definite increase is observed. For calculation of rates the material for the four years has been combined. The rates are thus based on 3653 Finnish males, 384 Finnish females, 776 Norwegian males and 198 Norwegian females.

TABLE III.—*Incidence of Primary Lung Cancer (Int. List No.* 162) *in Finland and Norway* 1954–57

| Residence | Number of new cases 1954 | 1955 | 1956 | 1957 | Total 1954–57 |
|---|---|---|---|---|---|
| | FINLAND, MALES | | | | |
| Helsinki | 108 | 123 | 114 | 144 | 489 |
| Provincial towns | 226 | 250 | 287 | 263 | 1026 |
| Rural areas | 448 | 518 | 562 | 610 | 2138 |
| The whole country | 782 | 891 | 963 | 1017 | 3653 |
| | FINLAND, FEMALES | | | | |
| Urban areas | 46 | 36 | 43 | 43 | 168 |
| Rural areas | 54 | 52 | 52 | 58 | 216 |
| The whole country | 100 | 88 | 95 | 101 | 384 |
| | NORWAY, MALES | | | | |
| Oslo | 82 | 67 | 68 | 57 | 274 |
| Provincial towns | 51 | 52 | 55 | 63 | 221 |
| Rural areas | 68 | 59 | 71 | 83 | 281 |
| The whole country | 201 | 178 | 194 | 203 | 776 |
| | NORWAY, FEMALES | | | | |
| Urban areas | 25 | 27 | 25 | 36 | 113 |
| Rural areas | 21 | 25 | 17 | 22 | 85 |
| The whole country | 46 | 52 | 42 | 58 | 198 |

FIG. 6.—Incidence of primary lung cancer (Int. List No. 162) in Finland and Norway. Age-specific rates per 100,000. Annual average 1954–57.

FIG. 7a.—Incidence of primary lung cancer (Int. List No. 162) in Finland and Norway, by residence. Age-specific rates per 100,000. Annual average 1954–57.

FIG. 7b.—Incidence of primary lung cancer (Int. List No. 162) in Finland and Norway, by residence. Age-specific rates per 100,000. Annual average 1954–57.

The age-specific rates shown in Fig. 6 clearly bring out the difference between the two countries and between the two sexes as regards the incidence of the disease. The difference is definitely more marked among males than among females. The shapes of the curves for the two sexes differ markedly. Whereas the rates for females increase up to the oldest age groups, the rates for males show a peak at about 65 years. This is a well-known observation and is considered consistent with the hypothesis of a difference in the aetiology of lung cancer in males and in females. In this context it should be pointed out that the maximum male rate is seen at a higher age in Finland than in Norway in the age groups 65–69 years and 60–64 years respectively. Random variations can hardly cause this discrepancy, but obviously it may be produced by differences

FIG. 8.—Incidence of primary lung cancer (Int. List No. 162) in Finland and Norway. Age-adjusted rates per 100,000. Annual average 1954–57. (Calculated by direct standardization. Total population of Finland and Norway as at January 1, 1956, used as standard population.)

in the diagnosis and reporting of the disease in the two countries. The age-specific rates in the capitals, provincial towns and rural areas (Fig. 7*a* and 7*b*) show very much the same picture as for the whole country. The only curve deviating from the usual pattern is that for males in rural Norway. This curve is rather flat and has no distinct peak being more similar to that for females.

To quantify the differences in incidence shown in Fig. 7–9 standardized rates have been calculated. The annual incidence rate per 100,000 among Finnish males is 57·6 and among Norwegian males 10·5. The corresponding figures for females are 4·4 and 2·3 (Fig. 8). The ratio between incidence rates in Finland and Norway is thus more than five for males and less than two for females.

For both sexes the urban/rural differences are greater in Norway than in Finland (Fig. 9). Furthermore the difference between capital and provincial towns is very slight in Finland. As a whole the ratios between incidence rates for urban/rural areas as well as capital/provincial towns are more than 50 per cent higher in Norway than in Finland. A future study of regional variations within each country may throw more light on this difference in the geographic distributions.

FIG. 9.—Incidence of primary lung cancer (Int. List No. 162) in Finland and Norway, by residence. Age-adjusted rates per 100,000. Annual average 1954–57. (Calculated as for Fig. 8.)

FIG. 10.—Basis of the diagnosis of new cases of lung cancer (Int. List No. 162) in Finland and Norway. Age-adjusted rates per 100,000. Annual average 1954–57. (Calculated as for Fig. 8. (Assuming that the age distribution in the four diagnosis categories is the same.)

For further evaluation of the data the cases have been classified into four groups according to the method of diagnosis (Fig. 10) :

(1) Histological examination of primary tumour or metastases.

(2) Autopsy, operation or endoscopy without histological examination with or without cytological examination.

(3) X-ray examination only.

(4) Clinical examination (including cases registered on the basis of death certificate only).

Compared to Norway a greater fraction of the cases in Finland is based on clinical examination or death certificate and a smaller fraction verified histologically. However, the rate of histologically verified male cases in Finland is about 2·5 times the total male rate in Norway.

For females the contrasts are smaller. As a whole the basis of the diagnosis is poorer among females. The female cases of lung cancer are older than the male cases. As the basis of diagnosis gets poorer with increasing age, one would expect a difference in this direction. The observed difference between the sexes in the diagnosis of lung cancer, however, can only partly be explained by the difference in age.

### SUMMARY AND CONCLUSIONS

Mortality and morbidity data on lung cancer are in close agreement with regard to variations within as well as between the two countries. The most striking observation in the present material is the considerable difference in the mortality and morbidity in Finnish and Norwegian males.

In the evaluation of such observations possible differences in diagnosis, classification and reporting of the disease in the two countries should be taken into account. Could the frequency of unrecognized lung cancer cases be so much higher in Norway or the frequency of cases erroneously classified as lung cancer be so much higher in Finland as to give an entirely distorted picture of the incidence of the disease in the two countries?

The great interest in lung cancer among Norwegian doctors and the good diagnostic facilities available seem to justify the assumption that the disease is not more frequently missed in Norway than in Finland. The alternative possibility that overdiagnosing might be more frequent in Finland cannot at present be excluded. A considerable fraction of " false positives " may be found among the cases not histologically verified, and almost 50 per cent of all lung cancer cases in Finland belong to this category, compared to slightly more than 10 per cent in Norway. Among the histologically verified cases one would, however, expect a negligible number only of " false positives ". This has been confirmed in a study of the histological types of lung tumours in Finland and Norway (Kreyberg and Saxén, 1961). It was established that practically all tumours represented primary lung tumours. Considering that the rate of histologically verified cases among Finnish males is more than twice the *total* rate among Norwegian males a ratio of two between incidence rates of the disease in the two countries may therefore be regarded as a minimum estimate. Even though the influence of diagnostic differences is impossible to quantify it seems reasonable to assume, however, that the ratio of about five as found in the present material gives a more correct picture.

The observed dissimilarities in the trend and present incidence of lung cancer in Finland and Norway, as regards sex, age and geographic variation, probably reflect differences in the exposure of the Finnish and Norwegian populations to factors causally related to lung cancer. However, the aetiological aspects will not be discussed here. At present the observations made will serve solely in the planning of future studies.

### REFERENCES

KREYBERG, L. AND SAXÉN, E.—(1961) *Brit. J. Cancer*, **15**, 211.
PEDERSEN, E. AND MAGNUS, K.—(1959) The Cancer Registry of Norway. Monograph No. 1.
SAXÉN, E. and KORPELA, A.—(1958) *Ann. Chir. Gyn. Fenn.*, **47**, Suppl. 79.
WORLD HEALTH ORGANIZATION—(1960) *Tech. Rep. Wld Hlth Org.*, No. 192.

APPENDIX TABLE I.—*Mortality from Cancer of the Lung in Finland and Norway*, 1934–58

FINLAND

| | Year | | | | | |
|---|---|---|---|---|---|---|
| | 36 | 39–41 | 44–46 | 49–51 | 54–56 | 57–58 |
| | | | MALES | | | |
| Helsinki: | | | | | | |
| Number of cases | 24 | 78 | 131 | 245 | 350 | 262 |
| Crude annual rate per 100,000 | 19·51 | 18·98 | 30·12 | 51·36 | 65·92 | 68·23 |
| Standardizing factor | 1·18 | 1·17 | 1·34 | 1·26 | 1·14 | 1·20 |
| Age-adjusted annual rate per 100,000 | 23·0 | 22·2 | 40·3 | 64·8 | 75·1 | 81·8 |
| Provincial towns: | | | | | | |
| Number of cases | 34 | 120 | 153 | 354 | 572 | 566 |
| Crude annual rate per 100,000 | 14·66 | 15·63 | 18·82 | 27·90 | 38·91 | 53·00 |
| Standardizing factor | 1·23 | 1·19 | 1·32 | 1·51 | 1·42 | 1·39 |
| Age-adjusted annual rate per 100,000 | 18·1 | 18·6 | 24·8 | 42·1 | 55·4 | 73·7 |
| Rural areas: | | | | | | |
| Number of cases | 103 | 443 | 665 | 1183 | 1517 | 1213 |
| Crude annual rate per 100,000 | 6·76 | 9·79 | 15·73 | 29·38 | 36·64 | 44·40 |
| Standardizing factor | 1·25 | 1·20 | 1·32 | 1·31 | 1·27 | 1·24 |
| Age-adjusted annual rate per 100,000 | 8·5 | 11·8 | 20·7 | 38·5 | 46·5 | 55·1 |
| The whole country: | | | | | | |
| Number of cases | 161 | 641 | 949 | 1782 | 2439 | 2041 |
| Crude annual rate per 100,000 | 8·57 | 11·23 | 17·33 | 30·87 | 39·72 | 48·78 |
| Standardizing factor | 1·24 | 1·20 | 1·32 | 1·35 | 1·29 | 1·27 |
| Age-adjusted annual rate per 100,000 | 10·7 | 13·4 | 22·8 | 41·7 | 51·2 | 62·0 |
| | | | FEMALES | | | |
| Urban areas: | | | | | | |
| Number of cases | 11 | 40 | 49 | 110 | 127 | 97 |
| Crude annual rate per 100,000 | 2·50 | 2·73 | 3·05 | 5·10 | 5·23 | 5·57 |
| Standardizing factor | 1·05 | 0·99 | 1·00 | 1·03 | 0·97 | 0·96 |
| Age-adjusted annual rate per 100,000 | 2·6 | 2·7 | 3·0 | 5·3 | 5·1 | 5·3 |
| Rural areas: | | | | | | |
| Number of cases | 17 | 73 | 120 | 207 | 178 | 114 |
| Crude annual rate per 100,000 | 1·14 | 1·62 | 2·72 | 4·99 | 4·21 | 4·09 |
| Standardizing factor | 1·09 | 1·06 | 1·09 | 1·08 | 1·03 | 1·01 |
| Age-adjusted annual rate per 100,000 | 1·2 | 1·7 | 3·0 | 5·4 | 4·3 | 4·1 |
| The whole country: | | | | | | |
| Number of cases | 28 | 113 | 169 | 317 | 305 | 211 |
| Crude annual rate per 100,000 | 1·45 | 1·90 | 2·81 | 5·03 | 4·58 | 4·66 |
| Standardizing factor | 1·08 | 1·05 | 1·06 | 1·06 | 1·01 | 0·99 |
| Age-adjusted annual rate per 100,000 | 1·6 | 2·0 | 3·0 | 5·3 | 4·6 | 4·6 |

### Appendix Table I—*contd.*

Norway

| | Year | | | | | |
|---|---|---|---|---|---|---|
| | 34–36 | 39–41* | 44–46 | 49–51 | 54–66 | 57–58 |
| | | | Males | | | |
| Oslo : | | | | | | |
| Number of cases | 8 | 11 | 44 | 133 | 212 | 126 |
| Crude annual rate per 100,000 | 2·22 | 4·51 | 11·64 | 22·50 | 34·14 | 29·72 |
| Standardizing factor | 1·09 | 1·10 | 0·93 | 0·92 | 0·82 | 0·82 |
| Age-adjusted annual rate per 100,000 | 2·4 | 5·0 | 10·8 | 20·7 | 28·0 | 24·4 |
| Provincial towns : | | | | | | |
| Number of cases | 18 | 25 | 38 | 96 | 143 | 132 |
| Crude annual rate per 100,000 | 2·36 | 4·79 | 4·49 | 10·92 | 15·28 | 20·75 |
| Standardizing factor | 1·36 | 1·03 | 1·01 | 0·96 | 0·92 | 0·92 |
| Age-adjusted annual rate per 100,000 | 3·2 | 4·9 | 4·5 | 10·5 | 14·1 | 19·1 |
| Rural areas : | | | | | | |
| Number of cases | 50 | 60 | 96 | 176 | 193 | 174 |
| Crude annual rate per 100,000 | 1·61 | 2·78 | 2·79 | 5·18 | 5·38 | 7·14 |
| Standardizing factor | 1·21 | 1·14 | 1·06 | 1·01 | 0·96 | 0·92 |
| Age-adjusted annual rate per 100,000 | 2·0 | 3·2 | 3·0 | 5·2 | 5·2 | 6·6 |
| The whole country : | | | | | | |
| Number of cases | 76 | 96 | 178 | 405 | 548 | 432 |
| Crude annual rate per 100,000 | 1·80 | 3·28 | 3·28 | 8·32 | 10·65 | 12·36 |
| Standardizing factor | 1·22 | 1·11 | 1·03 | 0·99 | 0·93 | 0·91 |
| Age-adjusted annual rate per 100,000 | 2·2 | 3·6 | 3·9 | 8·2 | 9·9 | 11·2 |
| | | | Females | | | |
| Urban areas : | | | | | | |
| Number of cases | 27 | 31 | 55 | 78 | 87 | 66 |
| Crude annual rate per 100,000 | 1·99 | 3·44 | 3·85 | 4·63 | 4·95 | 5·52 |
| Standardizing factor | 1·04 | 0·89 | 0·83 | 0·80 | 0·74 | 0·74 |
| Age-adjusted annual rate per 100,000 | 2·1 | 3·1 | 3·2 | 3·7 | 3·7 | 4·1 |
| Rural areas : | | | | | | |
| Number of cases | 40 | 45 | 94 | 98 | 94 | 60 |
| Crude annual rate per 100,000 | 1·30 | 2·11 | 2·79 | 3·00 | 2·74 | 2·58 |
| Standardizing factor | 1·08 | 1·01 | 0·96 | 0·92 | 0·88 | 0·85 |
| Age-adjusted annual rate per 100,000 | 1·4 | 2·1 | 2·7 | 2·8 | 2·4 | 2·2 |
| The whole country : | | | | | | |
| Number of cases | 67 | 76 | 149 | 176 | 181 | 126 |
| Crude annual rate per 100,000 | 1·51 | 2·50 | 3·10 | 3·55 | 3·49 | 3·58 |
| Standardizing factor | 1·07 | 0·97 | 0·92 | 0·88 | 0·83 | 0·81 |
| Age-adjusted annual rate per 100,000 | 1·6 | 2·4 | 2·9 | 3·1 | 2·9 | 2·9 |

* No data available for the year 1940.

APPENDIX TABLE III.—*Annual Age-specific and Age-adjusted Rates per* 100,000 *of New Cases of Primary Lung Cancer* (*Int. List No.* 162), *in Finland and Norway,* 1954–57, *According to Sex, Age and Residence*

| Residence | Age-group (years) 0–19 | –24 | –29 | –34 | –39 | –44 | –49 | –54 | –59 | –64 | –69 | –74 | 75– | Total crude rate | Total age-adjusted rate |
|---|---|---|---|---|---|---|---|---|---|---|---|---|---|---|---|
| FINLAND, MALES | | | | | | | | | | | | | | | |
| Helsinki | 0·4 | — | — | 1·9 | 11·5 | 28·4 | 59·8 | 160·6 | 285·6 | 385·6 | 518·9 | 456·6 | 353·3 | 69·5 | 83·6 |
| Provincial towns | 0·3 | 0·7 | 1·2 | 1·3 | 8·5 | 28·8 | 80·4 | 134·3 | 257·1 | 355·9 | 406·8 | 400·0 | 376·3 | 52·2 | 76·5 |
| Rural areas | 0·1 | 0·2 | 0·5 | 1·4 | 6·7 | 20·6 | 45·7 | 107·2 | 171·7 | 246·7 | 272·0 | 259·0 | 150·4 | 38·8 | 49·0 |
| The whole country | 0·2 | 0·3 | 0·6 | 1·4 | 7·7 | 23·6 | 56·2 | 119·6 | 203·2 | 283·7 | 319·5 | 299·3 | 197·5 | 43·6 | 57·6 |
| FINLAND, FEMALES | | | | | | | | | | | | | | | |
| Urban areas | — | 0·4 | 0·8 | 0·8 | 1·7 | 2·4 | 6·5 | 8·3 | 13·6 | 21·9 | 17·8 | 26·1 | 37·2 | 5·2 | 5·2 |
| Rural areas | — | 0·3 | — | — | 2·1 | 2·3 | 3·1 | 6·3 | 10·4 | 13·9 | 20·0 | 23·8 | 27·2 | 3·8 | 4·0 |
| The whole country | — | 0·3 | 0·3 | 0·3 | 1·9 | 2·3 | 4·5 | 7·1 | 11·7 | 17·0 | 19·2 | 24·6 | 30·6 | 4·3 | 4·4 |
| NORWAY, MALES | | | | | | | | | | | | | | | |
| Oslo | — | — | — | 1·5 | 3·0 | 21·0 | 27·9 | 44·4 | 111·4 | 143·5 | 126·5 | 101·9 | 92·3 | 32·9 | 26·7 |
| Provincial towns | — | — | 2·3 | — | 3·0 | 3·2 | 11·5 | 32·4 | 66·0 | 91·9 | 91·4 | 72·1 | 41·4 | 17·9 | 16·1 |
| Rural areas | — | 0·3 | 1·2 | 0·3 | 2·0 | 2·7 | 7·5 | 15·6 | 21·8 | 25·5 | 24·1 | 23·4 | 15·3 | 5·9 | 5·7 |
| The whole country | — | 0·2 | 1·3 | 0·4 | 2·3 | 5·2 | 10·7 | 23·2 | 44·0 | 56·0 | 50·4 | 41·5 | 27·5 | 11·3 | 10·5 |
| NORWAY, FEMALES | | | | | | | | | | | | | | | |
| Urban areas | 0·2 | — | 0·7 | 2·3 | 0·5 | 1·7 | 2·8 | 4·6 | 9·4 | 17·7 | 12·8 | 18·8 | 22·3 | 4·8 | 3·6 |
| Rural areas | — | — | — | — | 1·8 | — | 3·5 | 1·9 | 5·4 | 7·4 | 8·3 | 7·5 | 6·1 | 1·9 | 1·7 |
| The whole country | 0·0 | — | 0·2 | 0·8 | 1·3 | 0·6 | 3·2 | 3·0 | 7·0 | 11·4 | 10·0 | 11·8 | 11·8 | 2·9 | 2·3 |

APPENDIX TABLE II.—*Number of New Cases of Primary Lung Cancer (Int. List No. 162), in Finland and Norway, 1954–57, According to Sex, Age and Residence*

| Residence | Age group (years) | | | | | | | | | | | | | Tota |
|---|---|---|---|---|---|---|---|---|---|---|---|---|---|---|
| | 0–19 | –24 | –29 | –34 | –39 | –44 | –49 | –54 | –59 | –64 | –69 | –74 | 75– | |
| FINLAND, MALES | | | | | | | | | | | | | | |
| Helsinki | 1 | — | — | 1 | 6 | 16 | 34 | 71 | 100 | 101 | 85 | 45 | 29 | 489 |
| Provincial towns | 2 | 1 | 2 | 2 | 11 | 41 | 110 | 151 | 206 | 192 | 152 | 90 | [illegible]6 | 1026 |
| Rural areas | 3 | 1 | 2 | 5 | 20 | 67 | 152 | 299 | 392 | 437 | 374 | 245 | 141 | 2138 |
| The whole country | 6 | 2 | 4 | 8 | 37 | 124 | 296 | 521 | 698 | 730 | 611 | 380 | 236 | 3653 |
| FINLAND, FEMALES | | | | | | | | | | | | | | |
| Urban areas | — | 1 | 2 | 2 | 4 | 6 | 16 | 17 | 23 | 29 | 19 | 20 | 29 | 168 |
| Rural areas | — | 1 | — | — | 7 | 8 | 11 | 19 | 28 | 30 | 37 | 33 | 42 | 216 |
| The whole country | — | 2 | 2 | 2 | 11 | 14 | 27 | 36 | 51 | 59 | 56 | 53 | 71 | 384 |
| NORWAY, MALES | | | | | | | | | | | | | | |
| Oslo | — | — | — | 1 | 2 | 14 | 18 | 28 | 64 | 66 | 39 | 20 | 22 | 274 |
| Provincial towns | — | — | 2 | — | 3 | 3 | 10 | 25 | 46 | 53 | 40 | 23 | 16 | 221 |
| Rural areas | — | 1 | 4 | 1 | 7 | 9 | 21 | 42 | 53 | 51 | 38 | 28 | 26 | 281 |
| The whole country | — | 1 | 6 | 2 | 12 | 26 | 49 | 95 | 163 | 170 | 117 | 71 | 64 | 776 |
| NORWAY, FEMALES | | | | | | | | | | | | | | |
| Urban areas | 1 | — | 1 | 4 | 1 | 3 | 5 | 8 | 15 | 23 | 13 | 15 | 24 | 113 |
| Rural areas | — | — | — | — | 6 | — | 10 | 5 | 13 | 15 | 14 | 10 | 12 | 85 |
| The whole country | 1 | — | 1 | 4 | 7 | 3 | 15 | 13 | 28 | 38 | 27 | 25 | 36 | 198 |

APPENDIX TABLE IV.—*Basis of the Diagnosis of the New Cases of Primary Lung Cancer (Int. List No. 162) in Finland and Norway, 1954–57, according to Sex and Residence*

| Residence | Histological examination | | Autopsy, operation or endoscopy | | X-ray examination only | | Clinical examination or death certificate | | Total |
|---|---|---|---|---|---|---|---|---|---|
| | No. | % | No. | % | No. | % | No. | % | |
| FINLAND, MALES | | | | | | | | | |
| Helsinki | 377 | 77·1 | 33 | 6·7 | 65 | 13·3 | 14 | 2·9 | 489 |
| Provincial towns | 480 | 46·8 | 89 | 8·7 | 384 | 37·4 | 73 | 7·1 | 1026 |
| Rural areas | 811 | 37·9 | 160 | 7·5 | 942 | 44·1 | 225 | 10·5 | 2138 |
| The whole country | 1668 | 45·7 | 282 | 7·7 | 1391 | 38·1 | 312 | 8·5 | 3653 |
| FINLAND, FEMALES | | | | | | | | | |
| Urban areas | 72 | 42·9 | 13 | 7·7 | 61 | 36·3 | 22 | 13·1 | 168 |
| Rural areas | 56 | 25·9 | 10 | 4·7 | 114 | 52·7 | 36 | 16·7 | 216 |
| The whole country | 128 | 33·3 | 23 | 6·0 | 175 | 45·6 | 58 | 15·1 | 384 |
| NORWAY, MALES | | | | | | | | | |
| Oslo | 248 | 90·5 | 11 | 4·0 | 14 | 5·1 | 1 | 0·4 | 274 |
| Provincial towns | 173 | 78·3 | 14 | 6·3 | 34 | 15·4 | — | — | 221 |
| Rural areas | 207 | 73·7 | 26 | 9·2 | 48 | 17·1 | — | — | 281 |
| The whole country | 628 | 80·9 | 51 | 6·6 | 96 | 12·4 | 1 | 0·1 | 776 |
| NORWAY, FEMALES | | | | | | | | | |
| Urban areas | 90 | 79·6 | 7 | 6·2 | 16 | 14·2 | — | — | 113 |
| Rural areas | 49 | 57·6 | 18 | 21·2 | 18 | 21·2 | — | — | 85 |
| The whole country | 139 | 70·2 | 25 | 12·6 | 34 | 17·2 | — | — | 198 |